# Motor-cognitive interaction in adults with spina bifida: dual-task effects

**DOI:** 10.1038/s41393-025-01099-5

**Published:** 2025-08-21

**Authors:** Martina Bendt, Emelie Butler Forslund, Göran Hagman, Urban Ekman, Lucian Bezuidenhout, Hanna Johansson, Anders Rydström, Claes Hultling, Åke Seiger, Erika Franzén

**Affiliations:** 1 Karolinska Institutet, Department of Neurobiology, Care Science and Society, Division of Physiotherapy, Stockholm, Sweden; 2 Aleris Rehab Station, Stockholm, Sweden; 3 Karolinska Institutet, Department of Neurobiology, Care Science and Society, Division of Clinical Geriatrics, Stockholm, Sweden; 4 Karolinska Institutet, Department of Neurobiology, Care Science and Society, Division of Clinical Geriatrics, Aging Research Center, Stockholm, Sweden; 5 Karolinska University Hospital, Stockholm, Sweden; 6 Faculty of Community and Health Sciences, University of the Western Cape, Bellville, South Africa; 7 Karolinska University Hospital, Theme Women’s Health and Allied Health Professionals, Stockholm, Sweden; 8 Stockholms Sjukhem R and D unit, Stockholm, Sweden; 9 Spinalis Foundation, Stockholm, Sweden; 10 Sophiahemmet University College, Stockholm, Sweden; 11 Allied Health Professionals, Medical Unit Occupational Therapy and Physical Therapy, Karolinska University Hospital, Stockholm, Sweden

**Keywords:** Neurological disorders, Spinal cord diseases

## Abstract

**Study design:**

Cross-sectional design.

**Objectives:**

To study motor-cognitive interaction during walking while performing a cognitive task (dual-task [DT]) in ambulatory adults with spina bifida (SB).

**Setting:**

A specialized spinal cord center, The Spinalis clinic at Aleris Rehab Station Stockholm, Sweden.

**Methods:**

Assessments of muscle strength and ambulatory function were performed. Gait was assessed with a sensor-based system with/without the auditory Stroop. Timed-up-and-go (TUG) with/without a cognitive task was also assessed. Regarding cognitive function, episodic memory, executive function, and processing speed were assessed. The percentage of difference between single-task (ST) and dual-task (DT) was used to calculate the DT effect (DTE) as cost or benefit. Differences were analyzed with t-test and Wilcoxon’s signed rank test.

**Results:**

Forty-one persons were included, mean age 37 years (SD 12) and 20 (49%) were women. Thirty-four completed the DTE analysis. Seven could not perform the cognitive task and/or gait data and could not be registered. There was a DT cost on gait speed (4%), stride length (3%) and double support phase (3%), and cognitive function showed a cost of 3%. DTE for TUG was a 26% cost.

**Conclusion:**

We showed a DT cost on gait, possibly indicating fall risk during DT walking. The largest DT cost was seen during TUG with a cognitive task, indicating a valuable clinical test for motor-cognitive performance for adults with SB. This study is pioneering in that it increases our understanding of DT performance in ambulatory adults with SB which could facilitate development of targeted rehabilitation interventions and self-management strategies.

## Introduction

Spina bifida (SB) is a congenital malformation, with spinal cord involvement, associated with impaired muscle and sensory function in the lower limbs [1]. Persons with SB have a complex set of medical, physical and cognitive needs and they have an ongoing need for care and support throughout life [2]. The level and extent of spinal involvement define the ability to stand and walk [3] but ambulatory function is also influenced by other factors such as history of shunting, contractures in lower limbs and body mass index (BMI) [4–6]. Previous research has shown that adults with SB suffer from impaired gait and balance [2, 6–8]. In addition, they often have impaired cognitive function with reduced memory, attention, and executive functioning [1, 9]. which may have a negative impact on functional independence and quality of life [10]. However, they show high interindividual variability regarding both motor and cognitive function [1, 3, 6, 7, 10].

Activities in daily life often require dual-tasking (DT) such as simultaneous motor and cognitive tasks, e.g., walking while talking. Both impaired motor and cognitive function may interfere with performance of complex activities [11]. Assessing DT performance during gait in populations with neurological dysfunction has revealed walking difficulties not present during single task (ST) gait [12, 13]. This may comprise a risk of falls or other accidents in challenging situations such as busy environments.

Since dual-tasking is part of many activities in daily life, and adults with SB often have both motor and cognitive impairments it is important to explore this further. To our knowledge, DT performance in adults with SB has not yet been investigated. Also, the prioritisation between motor and cognitive tasks while performing a DT is an unexplored field in adults with SB. The prioritisation is dependent on motor and cognitive capacity as well as the demands of the tasks. Increased knowledge of motor-cognitive interaction can lead to more targeted interventions and better care for ambulatory adults with SB.

This study aimed to investigate DT effects (DTE), the motor-cognitive interaction, and explore potential patterns of prioritisation with regard to gait and cognition in ambulatory adults with SB.

## Methods

### Participants and procedure

This is a sub study from a large SB project at a specialized outpatient clinic for persons with spinal cord disorders, the Spinalis outpatient clinic at Aleris Rehab station in Stockholm, Sweden. It includes a near-total regional cohort of adults with SB.

**Fig. 1:**
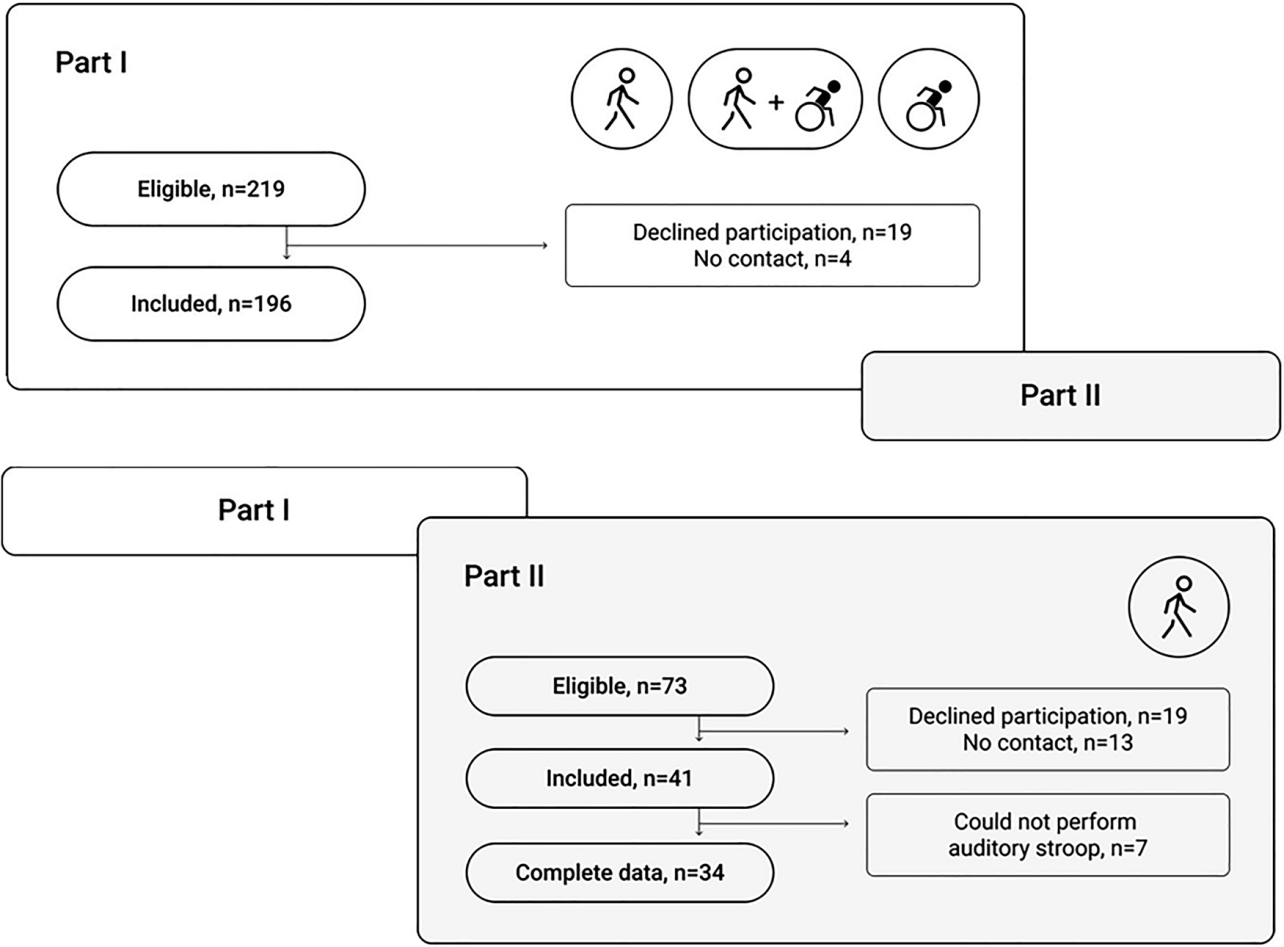
Flow chart of eligible and included participants in Part I and II.

In part I (Fig. 1) [2, 6] all adults with SB enrolled at the Spinalis outpatient clinic (*n* = 219) were consecutively invited to participate in conjunction with their regular follow-up program. In Part II [7] and present paper, those matching the criteria from Part I were asked to participate. In addition, individuals enrolled at the clinic after Part I were screened and invited to participate at the time of their scheduled follow-up or at their first attendance at the clinic. The inclusion criteria for Part II were adults with SB (age 18 to ≤65 years), regularly walking a minimum of 30 m (community or household ambulators as defined by Hoffer et al. [14]) with a level of MF 1–3 [3]. This level of muscle function (MF 3) was selected as it has been shown to be the critical level for functional walking in previous studies of adults with SB [3, 6]. Persons with MF 3 have full hip flexion and knee extensor strength but reduced knee flexion and only traces of hip extension and abduction and below knee muscles [3]. Exclusion criteria for Part II were additional diagnoses that affect ambulation and persons without any muscular impairments in the legs. As the aim was to explore motor cognitive DT effect in adults with SB, we had 65 years as an upper limit to avoid age- related changes in cognition [15]. Flow charts of eligible and the finally included participants in Parts I and II are presented in Fig. 1.

The near-total regional cohort of adults from Part I [2] was screened for participants, and all additional persons registered at the clinic after that study were invited to participate in conjunction with their regular SB follow-up program (Fig.  1). A cross-sectional design was used, and data collection was conducted on one occasion for around 1,5 h.

The data collection in part II was divided in two sections. Section A the cognitive assessments, was administered by a psychologist and section B including background data, clinical and physical assessments, was administrated by a physiotherapist with over 15 years of experience of adults with SB. Both Section A and B had a predefined order. Two participants were scheduled at the same time and the participant who arrived first started with Section A. The DTE during the Stroop test and the Timed Up and Go (TUG) with a simultaneous cognitive task, were considered primary outcomes.

### Section A, cognitive assessments

In brief, two episodic memory tests were used, the Rey Auditory Verbal Learning test (RAVLT total comprising five learning trials and RAVLT Delayed consisting of a 25 min delayed recall) [16] and non-the non-verbal Rey–Osterrieth Complex Figure Test (ROCF) [17]. Further, two verbal ability tests, the verbal fluency test (phonemic fluency FAS) from the Delis-Kaplan Executive Function System (D-KEFS) [18] and the vocabulary subtest from Wechsler Adult Intelligence Scale® fourth edition (WAIS‐IV) [19] were used.

Psychomotor speed and executive function were assessed by using the Trail Making Test (TMT) from D-KEFS [18]. From the WAIS‐IV, test attention and visuomotor processing speed was assessed with the Coding subtest, verbal reasoning with the similarities test, non-verbal problem solving with the Matrix Reasoning test, and visuospatial construction abilities with the Block Design test.

The included tests are commonly used in clinical practice to assess cognitive function in a wide range of persons with neurological diagnoses. The standard age-corrected norms were used to calculate the scaled scores on the WAIS-IV and D-KEFS tests [18], for the RAVLT the Van der Elst et al. norms [20] were used. For the ROCF results were transformed to scale scores (z [age and gender] × 3)+10 [21]. The scaled scores have a mean of 10 and one standard deviation is equal to a scaled score of 3, the range of average cognitive function can generally be found within one standard deviation from mean, which corresponds to scaled scores 7–13 (68% of the population).

### Section B, background data, clinical and physical assessments

A semi-structured interview was used to collect demographic data and information regarding orthoses and assistive devices. Functional walking ability was categorized as household or community walkers according to the criteria defined by Hoffer [14].

Sensory and motor functions were assessed according to the International Standards for Neurological Classification of Spinal Cord Injury [22]. Muscle strength in the lower extremities was assessed with a 0–5 graded manual muscle test according to Daniel’s and Worthingham, and participants were classified according to levels of MF 1–3 [3]. In persons with asymmetric muscle function the MF classification was based on the most affected leg, in order not to overrate their function and as this has been used in previous research within the field [2, 6, 23].

To assess walking, functional movement and balance the Timed Up and Go (TUG) was used [24]. It is a commonly used clinical test where the participant sits on a chair, rises, walks three meters, turn around and walks back to sit down again. TUG cognitive (TUGcog) with the additional task of counting backward by threes while performing the test was also used and the time difference was registered (TUG – TUGcog).

For gait analysis, a sensor-based motion analysis system (Opal, APDM Inc.) was used to collect temporal and spatial parameters of gait and Mobility Lab™ software was used for the analysis [25, 26]. Participants wore six synchronized inertial measurement unit sensors, placed at the sternum, waist, bilaterally on dorsal wrists, and on dorsal metatarsus respectively. Participants walked for 30 s in a 12 m corridor, first as a single task (ST) (just walking) and then as a DT with the Auditory Stroop (AS) as a concomitant cognitive task [27]. Participants were instructed to walk at their usual pace and performed practice trials first. Spatiotemporal parameters of gait have been previously reported in detail [7].

Standard deviations (SD) of the cadence, stride time, stride length, and double support were used as measures of variability. The most affected leg was used for participants with muscular asymmetry.

Stroop test is a commonly used cognitive test. It comes in different versions assessing executive functions (divided attention and inhibition) e.g. the brains ability to handle and process congruent and incongruent information. In this study the Auditory Stroop test (AS) [27] was used, which is considered a reliable test for executive functioning [27] and has been proven useful in clinical studies in people with, MS, Parkinson’s disease and healthy elderly [12, 13, 28]. Participants used headphones and were presented with four different stimuli - the words “high” or “low” in congruent or incongruent high or low pitch. The stimuli were randomly delivered för 30 s every 1.5–2 s. Participants were instructed to “respond as quickly and as accurately as possible” to the corresponding *pitch*. Participants were offered up to three practice trials of the AS task before data collection started. The cognitive stimuli were pseudo-randomised to present equal numbers of the four possible stimuli. The first stimulus was removed from every trial to ensure that a steady state gait was obtained. The AS was first performed as ST (when seated) and secondly as DT (during walking), three times each.

The AS responses were recorded and converted to waveform auditory files (WAV) (Audacity version 2.1.3 [29]), imported to MATLAB (version R2021b) [30] and a computer algorithm was created to determine all the reaction times. The mean and SD of RTs (used as a measure of intraindividual variability) were calculated in ST and DT conditions.

Absent answers were registered as incorrect answers and were not included in the reaction time analysis. Percentage correct answers were calculated for the ST and DT conditions, respectively. Trials with <60% accuracy were removed to ensure that the participants performed the task as intended [13].

The AS has been shown to be valid and reliable during DT gait assessments in healthy elderly persons and persons with other neurological diseases [12, 31].

### Dual-task analysis

Gait variables of the legs were averaged for each trial and an average of the trials was used in the analysis. Trials deviating >0.15 m/s were not considered representative and excluded. Dual-task effect (DTE, was calculated as the percentage of change between ST and DT conditions as follows: (DT–ST) / ST *100, [32]. A negative DTE value corresponds to a DT cost and a positive one corresponds to a DT benefit. Further, the DTEs on reaction time, accuracy and TUG were calculated in the same way as for the gait parameters. An overall DTE was calculated on cognition (DTEcog) to consider both reaction time and accuracy; (DTE reaction time+DTE accuracy)/2 as recommended [12].

Prioritization was calculated by subtracting the DTE on gait speed and double support phase from the DTE on cognition. To explore possible variations of prioritisation the sample was dichotomised according to the median of gait speed during ST and TMT-B results. Gait speed in ST was chosen as previously reported [7] since gait speed was slower in participants with MF3 compared to MF1 and MF2 [7]. The double support phase (the percentage of the gait cycle in which both feet are on the ground) was selected as a measure of gait stability [33]. TMT-B was chosen as it assesses a person’s cognitive flexibility, the ability to shift attention between tasks or information [18] which closely relates to the cognitive functions involved in the dual-task gait tests.

### Statistical analyses

Analyses were performed using SPSS version 28 (IBM Corp., Armonk, NY, USA).

Descriptive data were presented as numbers and proportions. Normality was analyzed with the Shapiro-Wilks test, and by visual inspection of QQ-plots and histograms. Mean and SD were used for normally distributed variables, while median (Md) and interquartile ranges (IQR) were used for variables with non-normal distribution.

The independent t-test was used to assess between-group differences. A dependent t-test was used to assess the intraindividual difference between performance in ST and DT, and the effect size was calculated using Cohen’s d. Wilcoxon’s signed rank test was used for variables with a non-normal distribution and the effect size was calculated by using the z value $(r=z/\surd 2n)$, where *r* = effect size and *n* = number of participants. Spearman rank correlation analysis was used for correlations and were classified according to Dancey and Reidy [34]. Statistical significance was determined at *p* ≤ 0.05.

## Results

The 41 included persons had a mean age of 37 years (SD 12) and 20 (49%) were women (Table 1). Twenty-three persons (68%) were community ambulators and 11 (32%) were household ambulators. Three persons used walking aids (canes or crutches) and 19 (46%) used orthoses, of whom 13 used ankle-foot orthoses. Seven participants had motor asymmetry in the legs. Mean gait speed was 0.96 m/s (SD 0.21). The level of cognitive functioning was within the normative expected average range based on their age. However, the tests for executive function and mental speed (TMT-A, TMT-B and verbal fluency) were in the lower range of this span.

**Table 1:** Descriptive characteristics and results of cognitive tests displayed for those included and excluded in the dual-task effect (DTE) analysis with p-values and effect sizes.

	Total sample	Included in DTE analysis	Not included in DTE analysis	*P* value	Effect size
Participants, n (%)	41	34	7		
Sex, Women, n (%)	21(51)	17 (50)	3 (43)		
Age, mean (SD)	37 (12)	38 (12)	31 (12)	0.200	0.541
Min-max	19–59	25–59	19–50		
Length (cm) mean (SD)	162 (10)	162 (9.5)	160 (13)	0.628	0.203
Min-max	141–185	150–185	141–173		
Weight (kg), mean (SD)	68.0 (32)	74 (22)	73 (19)	0.859	0.079
Min-max	47–145	47–145	54–107		
BMI, mean (SD)	28.1 (6.3)	27.9 (6.5)	28.9 (5.3)	0.729	−0.155
Min-max	18.6–44.0	18.6–44.0	21.2–37.0		
Hydrocephalus, n (%)	23 (56)	17 (50)	6 (86)	0.112	
Contractures lower limb^a^, n (%)	39 (95)	33 (97)	6 (86)	0.204	
Neurological category^b^, n (%)					
L1 - L5AIS A	34 (83)	28 (82)	6 (86)		
L3 AIS C	1 (2)	1 (3)	—		
T11 - L3 AIS D	6 (15)	5 (15)	1 (14)		
Muscle function group, n (%)					
MF1^c^	3 (7)	1 (3)	2 (29)		
MF2^d^	12 (29)	12 (35)	—		
MF3^e^	26 (63)	21 (62)	5 (71)		
Ambulatory level, n (%)					
Community^f^	27 (66)	23 (68)	4 (57)		
Household^g^	14 (34)	11 (32)	3 (43)		
TUG s, mean (SD)	9.6 (2.4)	10.3 (2.3)	9.8 (2.1)	0.64	0.196
TUGcog s, mean (SD)	13.2 (6.0)	14.4 (5.8)	11.2 (4.9)	0.47	0.328
TUGcog failed to perform, n (%)	15 (37)	12 (35)	3 (43)		
Cognition^h^, mean (SD)					
Block Design	8.03 (2.78)	8.30 (2.80)	6.71 (2.43)	0.16	0.58
Similarities	8.10 (2.80)	8.32 (2.59)	7.0 (3.70)	0.39	0.47
Matrix Reasoning	8.65 (3.76)	9.09 (3.74)	6.57 (3.36)	0.11	0.68
Vocabulary	7.40 (2.12)	7.60 (2.15)	6.43 (1.81)	0.16	0.56
Coding	7.80 (3.17)	8.12 (3.27)	6.29 (2.21)	0.09	0.58
TMT-A^i^	6.88 (3.79)	7.39 (3.70)	4.43 (3.46)	0.07	0.81
TMT-B^j^	7.48 (3.32)	7.85 (3.35)	5.71 (2.75)	0.10	0.65
Verbal Fluency (FAS^k^)	9.95 (4.65)	10.91 (4.33)	5.43 (3.41)	0.003	1.30
RAVLT^l^ Total	9.54 (3.44)	10.39 (3.03)	5.46 (2.21)	<0.001	1.69
RAVLT Delayed	9.40 (3.34)	9.83 (3.0)	7.29 (4.33)	0.18	0.79
ROCF^m^ Copy	8.13 (4.07)	8.52 (4.14)	6.29 (3.40)	0.16	0.55
ROCF Delayed Recall	7.83 (3.71)	8.36 (3.49)	5.29 (3.90)	0.09	0.87

*DTE* dual-task effect, *BMI* body mass index, *MF* muscle function, *TUG* timed up and go, *TUGcog* TUG with dual-task, count backwards by threes.

^a^≥20^°^ including arthrodesis.

^b^AIS ASIA (American spinal injury association) impairment scale.

^c^With weakness in foot intrinsic muscles and plantar flexors, grade 4–5.

^d^With foot plantar flexion, grade ≤3, knee flexion grade ≥3, hip extension and/or hip abduction, grade ≤2–3.

^e^With hip flexion and knee extension, grade 4–5, knee flexion, grade ≤3, and only traces of hip extension, hip abduction, and below-knee muscles.

^f^Walking both in- and outdoors might use a wheelchair for long trips out of the community.

^g^Household ambulators mostly using a wheelchair for activities in the community and walking indoors

^h^The scaled scores have a mean of 10 and one standard deviation is equal to a scaled score of 3, the range of average cognitive function can generally be found within one standard deviation from mean, which corresponds to scaled scores 7–13.

^i^Trail making test (number-sequencing) from D-KEFS (Delis-Kaplan executive function system).

^j^Trail making test (number-letter switching conditions) from D-KEFS (Delis-Kaplan executive function system).

^k^Using the letter F, A and S.

^l^Rey auditory verbal learning test.

^m^Non-verbal Rey–Osterrieth complex figure test.

Of the 41 persons (Fig. 1, Table 1), seven (mean age 31 years [SD 12], three women) could not be included in the DTE analysis since they failed to perform the AS task. For three of those, the gait analysis system could not register enough gait cycles due to abnormal gait pattern. For the seven participants not able to perform the AS task, we found that the cognitive level was lower than for the sample of 34 with regard to the verbal fluency test (*p* = 0.003)] and the Rey Auditory Verbal Learning test (*p* ≤ 0.001).

Thereby there were 34 participants included in the DTE analysis (Table 1) with a mean age of 38 years (SD 12) and 17 were women.

### Dual-task effect – gait analysis

The mean number of analyzed gait cycles was 46 (SD 8.5) in the ST condition and 48 (SD 9.6) in the DT condition. For two participants one trial in ST deviated >0.15 m/s from the others and was therefore excluded. During DT walking, gait speed was reduced by 0.06 m/s (*p* = 0.003, a DT cost of 4%) and the stride length was reduced by 5 cm (*p* = 0.001, a DT cost of 3%), (Table 2). Cadence was reduced by 2 steps/min (*p* = 0.009, a DT cost of 3%) while the double support phase increased by 1% of the gait cycle (*p* < 0.001, a DT cost of 3%), Table 2. Thoracic rotation showed a small increase in range of motion by 2 degrees (*p* = 0.049) while the other trunk parameters were unchanged. There was a 12% (*p* = 0.029) DT cost on variability of cadence, whereas no DTE were seen in variability of stride time, stride length or proportion of time with double support.

**Table 2:** Dual-task effect for gait characteristics and cognitive outcome in Single-task (ST) and Dual-task (DT) condition for 34 participants.

	ST	DT	Diff ST-DT	*P*-value	Effect size	DTE (%)
Gait characterics						
Lower Limb						
Gait speed^a^ (m/s)	0.96 (0.21)	0.90 (0.22)	0.06	0.003	0.561	−4.33 (9.72)^b^
Cadence^c^ (steps/min)	106.30 (11.23)	103.52 (12.76)	2.78	0.009	0.478	−2.47 (5.35)^b^
Gait cycle duration^d^ (s)	1.14 (0.13)	1.15 (0.16)^b^	−0.01	0.006	−0.475	−2.54 (5.50)^b^
Stride time^e^ (s)	0.58 (0.06)	0.58 (0.08)^b^	−0.01	0.024	−0.388	−2.63 (6.12)^b^
Swing phase^f^ (% GCT)	36.94 (3.02)	36.35 (3.29)	0.59	0.001	0.613	−1.28 (2.55)^b^
Stance phase^g^ (% GCT)	63.06 (3.02)	63.65 (3.29)	−0.59	0.001	−0.611	−0.77 (1.30)^b^
Double support^h^ (% GCT)	26.74 (5.62)	27.71 (6.18)	−0.97	<0.001	−0.650	−2.94 (4.43)^b^
Stride length^i^ (m)	1.08 (0.18)	1.03 (0.18)	0.05	0.001	0.598	−3.37 (7.19)^b^
Lateral Step variability^j^ (cm)	4.90 (1.65)	4.82 (1.57)	0.08	0.536	0.109	−1.35 (23.56)
Circumduktion^k^ (cm)	3.08 (1.76)	3.07 (1.68)	0.73	0.701	0.066	−0.44 (22.98)^b^
Elevation at midswing^l^ (cm)	2.19 (1.09)	1.97 (1.09)	0.22	<0.001	0.711	−10.39 (22.76)
Variability						
SD of cadence (steps/min)	3.16 (1.60)^b^	2.73 (1.54)^b^	0.43	0.029	−0.350	−12.13 (37.68)^b^
SD of stride time (s)	0.02 (0.01)^b^	0.02 (0.01)^b^	0.00	0.099	−0.252	−14.29 (39.41)^b^
SD of double support (% GCT)	1.34 (0.69)^b^	1.29 (0.80)^b^	0.05	0.918	−0.016	−0.51 (29.26)^b^
SD of stride length (m)	0.04 (0.02)^b^	0.04 (0.02)^b^	0.00	0.216	−0–198	−7.42 (36.40)^b^
Lumbar – RoM (◦)						
Frontal (lateral sway)^m^	11.13 (4.97)	10.74 (4.76)	0.39	0.204	0.222	−1.12 (12.59)
Sagittal (forward/backward tilt)^n^	8.30 (5.94)^b^	8.11 (5.71)^b^	0.19	0.467	−0.116	0.52 (14.36)
Transverse (rotation)^o^	15.81 (12.30)^b^	14.85 (12.85)^b^	0.96	0.330	−0.156	0.44 (10.09)
Thoracic – RoM (◦)						
Frontal (lateral sway)^p^	14.86 (14.88)^b^	14.85 (14.96)^b^	0.01	0.533	−0.100	0.61 (11.93) ^b^
Sagittal (forward/backward tilt)^q^	7.34 (3.55)^b^	7.14 (4.87)^b^	0.20	0.388	−0.138	−1.07 (12.41)
Transverse (rotation)^r^	12.23 (6.32)	13.71 (7.60)	−1.48	0.049	−0.357	4.28 (14.15)
Cognition (tot)						−3.31
Reaction time						
All stimuli (s)	0.97 (0.13)	1.01 (0.14)	−0.04	0.138	−0.252	−4.90 (16.02)
Variability						
SD of RT	0.20 (0.13)^b^	0.22 (0.15)^b^	0.02	0.177	−0.216	−11.11 (79.91)^b^
Accuracy^b^						
Total mean (SD) (%)	97.76 (3.62)	96.03 (5.37)	−1.71	—	—	–1.71 (5.35)
Total median (IQR) (%)	100 (2.63)^b^	97.30 (6.08)^b^	−2.70	0.021	−0.370	—

A negative DT effect (DTE) value indicates a DT cost, and a positive DTE indicates a DT benefit.

*Diff* difference, *RoM* range of motion, *RT* reaction time, *SD* standard deviation.

^a^The forward speed of the subject, measured as the forward distance travelled during the gait cycle divided by the gait cycle duration.

^b^Non-normal distribution presented with median (IQR).

^c^The number of steps per minute, counting steps made by both feet.

^d^The duration of a full gait cycle, measured from the left foot’s initial contact to the next initial contact of the left foot.

^e^The duration of a step, stride time (The APDM calls it step duration), measured as the period from initial contact of one foot to the next initial contact of the opposite foot.

^f^The percentage of the gait cycle in which the foot is not on the ground.

^g^The percentage of the gait cycle in which the foot is on the ground.

^h^The percentage of the gait cycle in which both feet are on the ground.

^i^The forward distance travelled by a foot during a gait cycle.

^j^In a series of 3 consecutive foot placements of the same foot, the variability of perpendicular deviations of the middle foot placement from the line connecting the first and third.

^k^The distance that the foot travels perpendicular to forward movement while swinging forward during an individual stride.

^l^The hight, clearance, of the foot sensor measured at mid swing, relative to its start position while standing.

^m^The angular range of the lumbar spine in the coronal plane (roll).

^n^The angular range of the lumbar spine in the sagittal plane (pitch).

^o^The angular range of the lumbar spine in the transverse plane (yaw).

^p^The angular range of the thoracic spine in the coronal plane (roll).

^q^The angular range of the thoracic spine in the sagittal plane (pitch).

^r^The angular range of the thoracic spine in the transverse plane (yaw).

### Dual-task effect – auditory stroop

Seven trials with <60% accuracy were excluded from the AS test. Thirteen participants (38%) had 100% correct responses. Of all 2613 responses 79 (3%) were incorrect, and of those 73% were after an incongruent stimulus. There was no difference in reaction time between ST and DT, but accuracy decreased during DT (*p* = 0.021), see Table 2. The total DTE for cognition (i.e., reaction time and accuracy) showed a mean DT cost of 3.3% (SD 9.0).

### Dual-task effect – timed up and Go cognitive

Fifteen participants out of 41 failed to perform TUGcog. They either did not manage to perform the cognitive task (stopped talking when walking) or did not return to the chair as intended. There was no correlation (r_s_ = 0.12, *p* = 0.47) between those who failed to perform TUGcog and the participants not able to perform the AS task (*n* = 7). Mean time for TUG was 10.2 s (SD 2.3) and for TUGcog 13.2 s (SD 5.4), and the DTE for TUG showed a median DT cost of 26%.

### Patterns of prioritisation

Analyses of possible prioritisation in the total sample did not show any significant results for either the motor or the cognitive task, see Table 3. Neither did exploratory analysis, dividing the sample according to higher/lower gait speed and higher/lower psychomotor capacity (TMT-B), show any significant differences, see Table 3.

**Table 3:** Possible prioritization (cognition vs gait) displayed for all participants and for participants according to higher/lower gait speed and higher/lower psychomotor function capacity (cognitive function).

	Cognition-Gait speed	*p* value	Cognition -Double support phase	*p* value
Total group	Cognition 1.5%	0.331	Cognition 0.7%	0.712
Lower gait speed^a^	Gait (0.26%)	0.942	Gait (0.79%)	0.801
Higher gait speed^b^	Cognition (4.60%)	0.093	Cognition (2.15%)	0.329
*p* value	0.275		0.436	
Lower psychomotor capacity^c^	Cognition (5.75%)	0.198	Cognition (3.50%)	0.331
Higher psychomotor capacity^d^	Gait (0.66%)	0.739	Gait (1.53%)	0.383
*p* value	0.186		0.214	

^a^Gait speed ST <Md (0.95 m/s) (*n* = 17).

^b^Gait speed ST >Md (0.95 m/s) (*n* = 17).

^c^TMT-B <Md (8) (*n* = 15).

^d^TMT-B ≥Md (8) (*n* = 19).

## Discussion

This is to our knowledge the first study analyzing motor cognitive interaction in ambulatory adults with SB. The results are based on an extensive physical and neuropsychological test battery including a sample from a near total regional cohort of adults with SB. Some of the main findings were the difficulties to perform the TUG cog, and the DT cost of 2–4% in most gait parameters. The DTE’s might seem small, but in persons with severely impaired gait it might potentially be a clinically relevant difference. Slower gait speed and shorter steps in people already walking slowly might increase the risk of falls which are known to be common in ambulatory persons with spinal cord injury [35], a diagnosis with similar sensorimotor challenges.

The DTE on reaction time showed a considerably smaller cost than in a study on persons with Parkinson’s disease [13] (with a mean age of 71), but similar to a study of persons with mild (mean age 46 years) or moderate MS (mean age of 54 years) [28]. Healthy controls (mean age 49 years) did not have any DTE in response time. The Parkinson sample was in general almost 40 years older which may partly explain the difference in DTE on the cognitive task, apart from the cognitive differences due to the diagnoses. Nevertheless, the cognitive assessments in these studies were somewhat different and comparisons should be interpreted with caution. Also, those diseases are progressive in nature which must be considered. However, the above-mentioned studies [13, 28] included only incongruent stimuli indicating that adults with SB had a larger DTE on reaction time as this study included both congruent and incongruent stimuli. On the other hand, regarding accuracy, there was a small but significant cost for the SB group while there was no change for the other diagnoses [13, 28]. The SB sample seemed to respond faster but less accurately.

The 4% DT cost on gait speed in our study was almost comparable to persons with Parkinson’s disease and moderate MS, while persons with mild MS and healthy controls showed less DT costs [13, 28].

More than one out of three (*n* = 12, 37%) of those with complete data from the DTE analysis (Mobility Lab^TM^ gait analysis and AS) did not manage the TUGcog and a large DT cost of 26% was seen on those managing the test. This was a result of difficulties both in performing the subtraction and the walking task, thereby indicating the challenge of motor-cognitive performance. TUG is a complex motor task constituting sit-to-stand, walking, turning and sitting down again. Adding a cognitive task corresponds to many daily activities that are performed simultaneously such as walking and talking. Our results support TUGcog as a valuable clinical DT assessment tool and easy to administer in clinical work. Interestingly, we did not find any correlation between the performance of TUGcog and the AS during DT. This might be explained by the difference in cognitive tasks i.e. TUGcog with subtraction and the AS with congruent and incongruent stimuli. Also, by a partly different motor assessment, were walking straight forward during AS would seem easier compared to the different movements included in TUG.

The effects on gait parameters, TUGcog and cognition may imply impaired gait stability [33]. Difficulties with motor-cognitive performance in daily life may result in falls and/or cognitive challenges such as remembering what to buy when walking in a busy environment in a grocery store.

Regarding measurements, this DT setup has not previously been used for people with SB, but equivalent setups have been used for people with Parkinson’s disease [13] and MS [28]. The Mobility Lab software has previously been widely used to assess gait performance under ST and DT walking conditions in community-dwelling older adults [36], and in people with different neurological conditions of different ages [28, 37, 38].We used a sensor-based gait analysis system and we regard such systems advantageous as they may provide more ecologically valid data and can be used in clinical settings where patients are more comfortable compared to laboratory settings.

The AS task was too challenging for six participants; five consequently replied with the word but not the pitch in the DT condition and one did not manage >60% accuracy (i.e. above the level of chance) during the AS and was therefore excluded. The relatively large number of excluded persons (*n* = 6, 17%) from the DTE analysis may suggest that assessing DT in adults with SB requires adjustment with regard to both the gait analysis and the cognitive task in future studies. On the other hand, the set up was appropriate for the vast majority (83%) of our participants.

In general, the study participants showed a cognitive function in the lower end of the normal range, and the seven participants not included in the DTE analysis showed even lower results in the Verbal Fluency and the RAVLT test. These tests relate to executive function and episodic memory, with the Verbal Fluency test being more specifically related to the ability to regulate thoughts and behavior towards a specific goal or task [18], and the RAVLT more related to the ability to encode, store and recover verbal memory information [16]. Therefore, the results may explain why these persons could not manage the AS task as the outcome is predicted by verbal working memory and processing speed.

As this is a new group for DT testing, we have used studies with persons with Parkinsons disease and MS for comparisons, but we acknowledge that they differ with regard to their progressive nature, although they share some motor and cognitive involvement. To enable a better understanding of motor-cognitive interaction of the entire SB population future studies should also include wheelchair users. A high DT cost could possibly increase the risk of falls and challenge motor-cognitive performance in daily life also for wheelchair users, as the need of wheelchair usage has been shown to be associated with lower cognitive capacity compared to that of ambulatory persons [6]. Knowledge regarding falls in persons with SB is lacking, but falls have been shown to be common in wheelchair users with acquired spinal cord injury [39]. Life-long follow-up programs for persons with SB are of great importance including assessment of gait, balance and cognitive status. Training and updating of technical aids as well as strategies to address challenges should be proposed. Above all, future studies should explore whether DT performance can be improved by intervention strategies.

In conclusion, ambulatory adults with SB showed a DT cost on most of the gait parameters, possibly indicating a risk of falling while performing DT. For cognitive DT performance, we found no change in RT but a reduction in accuracy indicating that the participants answered quickly but inaccurately. There was no clear pattern of prioritisation towards either motor or cognitive performance during DT. As the largest DT cost was seen during TUG, we consider it a valuable clinical DT test for adult persons with SB. These results contribute to a better understanding of DT performance which should be considered when designing rehabilitative interventions and self-management strategies in order to improve DT performance in daily life of ambulatory adults with SB.

## Data Availability

Due to Swedish and EU personal data legislation, the data set is not publicly available but is available from the corresponding author upon appropriate request. Any sharing of data will be regulated via a data transfer and user agreement with the recipient.

## References

[1] Copp AJ, Adzick NS, Chitty LS, Fletcher JM, Holmbeck GN, Shaw GM. Spina bifida. Nat Rev Dis Primers. 2015;1:15007. 27189655 10.1038/nrdp.2015.7 PMC4898641

[2] Bendt M, Gabrielsson H, Riedel D, Hagman G, Hultling C, Franzen E, et al. Adults with spina bifida: a cross-sectional study of health issues and living conditions. Brain Behav. 2020;10:e01736. 32633090 10.1002/brb3.1736 PMC7428499

[3] Bartonek A, Saraste H. Factors influencing ambulation in myelomeningocele: a cross-sectional study. Dev Med Child Neurol. 2001;43:253–60. 11305403 10.1017/s0012162201000482

[4] Davis WA, Zigler CK, Crytzer TM, Izzo S, Houtrow AJ, Dicianno BE. Factors associated with ambulation in myelomeningocele: a longitudinal study from the national spina bifida patient registry. Am J Phys Med Rehabil. 2020;99:586–94. 32209832 10.1097/PHM.0000000000001406 PMC8968579

[5] Tita AC, Frampton JR, Roehmer C, Izzo SE, Houtrow AJ, Dicianno BE. Correlation between neurologic impairment grade and ambulation status in the adult spina bifida population. Am J Phys Med Rehabil. 2019;98:1045–50. 30932916 10.1097/PHM.0000000000001188 PMC8246589

[6] Bendt M, Seiger Å, Hagman G, Hultling C, Franzen E, Forslund EB. Adults with spina bifida: ambulatory performance and cognitive capacity in relation to muscle function. Spinal Cord. 2022;60:122–8. 34262127 10.1038/s41393-021-00658-w

[7] Bendt M, Forslund EB, Hagman G, Hultling C, Seiger Å, Franzén E. Gait and dynamic balance in adults with spina bifida. Gait Posture. 2022;96:343–50. 35820238 10.1016/j.gaitpost.2022.06.016

[8] Lundberg Larsen K, Maalen-Johansen IK, Rennie L, Lidal IB. Gait function in adults aged 50 years and older with spina bifida. Arch Phys Med Rehabil. 2002;102:702–8. 10.1016/j.apmr.2020.10.118 33166524

[9] Dennis M, Landry SH, Barnes M, Fletcher JM. A model of neurocognitive function in spina bifida over the life span. J Int Neuropsychol Soc. 2006;12:285–96. 16573862 10.1017/S1355617706060371

[10] Sachdeva S, Kolarova MZ, Foreman BE, Kaplan SJ, Jasien JM. A systematic review of cognitive function in adults with spina bifida. Dev Neurorehabil. 2021;24:569–82. 33872130 10.1080/17518423.2021.1907813

[11] Dennis M, Barnes MA. The cognitive phenotype of spina bifida meningomyelocele. Developmental Disabil Res Rev. 2010;16:31–9. 10.1002/ddrr.89 PMC2924202 20419769

[12] Strouwen C, Molenaar EA, Keus SH, Munks L, Bloem BR, Nieuwboer A. Test-retest reliability of dual-task outcome measures in people with parkinson disease. Phys Ther. 2016;96:1276–86. 26847010 10.2522/ptj.20150244

[13] Johansson H, Ekman U, Rennie L, Peterson DS, Leavy B, Franzen E. Dual-task effects during a motor-cognitive task in parkinson’s disease: patterns of prioritization and the influence of cognitive status. Neurorehabil Neural Repair. 2021;35:356–66. 33719728 10.1177/1545968321999053 PMC8073879

[14] Hoffer MM, Feiwell E, Perry R, Perry J, Bonnett C. Functional ambulation in patients with myelomeningocele. J Bone Joint Surg Am. 1973;55:137–48. 4570891

[15] Zimmerman B, Rypma B, Gratton G, Fabiani M. Age-related changes in cerebrovascular health and their effects on neural function and cognition: a comprehensive review. Psychophysiology. 2021;58:e13796 10.1111/psyp.13796 33728712 10.1111/psyp.13796 PMC8244108

[16] Schmidt M. Rey auditory and verbal learning test: a handbook. Los Angeles: Western Psychological Services; 1996.

[17] Meyers JE, Meyers KR. Rey complex figure test and recognition trial professional manual. Odessa, FL: Psychological Assessment Resources; 1995a.

[18] Delis DKE, Kramer J. The Delis-Kaplan executive function system: examiner’s manual. San Antonio, TX: The Psychological Corporation; 2001.

[19] Weschler D. Wechsler adult intelligence scale. 4th ed. San Antonio, TX: Stat Solut; 2008. pp. 1–3.

[20] Van der Elst W, van Boxtel MP, van Breukelen GJ, Jolles J. Rey’s verbal learning test: normative data for 1855 healthy participants aged 24–81 years and the influence of age, sex, education, and mode of presentation. J Int Neuropsychol Soc. 2005;11:290–302. 15892905 10.1017/S1355617705050344

[21] Fastenau PS, Denburg NL, Hufford BJ. Adult norms for the rey-osterrieth complex figure test and for supplemental recognition and matching trials from the extended complex figure test. Clin Neuropsychol. 1999;13:30–47. 10937646 10.1076/clin.13.1.30.1976

[22] Kirshblum SC, Burns SP, Biering-Sorensen F, Donovan W, Graves DE, Jha A, et al. International standards for neurological classification of spinal cord injury (revised 2011). J Spinal Cord Med. 2011;34:535–46. 22330108 10.1179/204577211X13207446293695 PMC3232636

[23] Bartonek A, Saraste H, Knutson LM. Comparison of different systems to classify the neurological level of lesion in patients with myelomeningocele. Dev Med Child Neurol. 1999;41:796–805. 10.1017/S0012162299001607 10619277 10.1017/s0012162299001607

[24] Podsiadlo D, Richardson S. The timed “Up & Go”: a test of basic functional mobility for frail elderly persons. J Am Geriatr Soc. 1991;39:142–8. 1991946 10.1111/j.1532-5415.1991.tb01616.x

[25] Mancini M, King L, Salarian A, Holmstrom L, McNames J, Horak FB. Mobility lab to assess balance and gait with synchronized body-worn sensors. J Bioeng Biomed Sci. 2011. 10.4172/2155-9538.S1-007. 10.4172/2155-9538.S1-007 PMC4062543 24955286

[26] Fang X, Liu C, Jiang Z. Reference values of gait using APDM movement monitoring inertial sensor system. R Soc Open Sci. 2018;5:1708–18. 10.1098/rsos.170818 PMC5792878 29410801

[27] Kestens K, Degeest S, Miatton M, Keppler H. An Auditory stroop test to implement in cognitive hearing sciences: development and normative data. Int J Psychol Res. 2021;14:37–51. 10.21500/20112084.5118 PMC8794330 35096355

[28] Wallin A, Franzen E, Bezuidenhout L, Ekman U, Piehl F, Johansson S. Cognitive-motor interference in people with mild to moderate multiple sclerosis, in comparison with healthy controls. Mult Scler Relat Disord. 2022;67:104181. 36174259 10.1016/j.msard.2022.104181

[29] Audacity. Accessed February 15, 2021. www.coursehero.com/file/74487874/READMEtxt/

[30] MATLAB. Version R2017b. MathWorks Inc; 2017.

[31] Tsang CSL, Chong DYK, Pang MYC. Cognitive-motor interference in walking after stroke: test-retest reliability and validity of dual-task walking assessments. Clin Rehabil. 2019;33:1066–78. 30722681 10.1177/0269215519828146

[32] Kelly VE, Janke AA, Shumway-Cook A. Effects of instructed focus and task difficulty on concurrent walking and cognitive task performance in healthy young adults. Exp Brain Res. 2010;207:65–73. 20931180 10.1007/s00221-010-2429-6 PMC3058115

[33] Cromwell RL, Newton RA. Relationship between balance and gait stability in healthy older adults. J Aging Phys Act. 2004;12:90–100. 15211023 10.1123/japa.12.1.90

[34] Dancey CP, Reidy J. Statistics without maths for psychology. Harlow. Pearson Education Limited; 2007.

[35] Jørgensen V, Butler Forslund E, Opheim A, Franzén E, Wahman K, Hultling C, et al. Falls and fear of falling predict future falls and related injuries in ambulatory individuals with spinal cord injury: a longitudinal observational study. J Physiother. 2017;63:108–13. 28343914 10.1016/j.jphys.2016.11.010

[36] Fang X, Jiang Z. Three-dimensional thoracic and pelvic kinematics and arm swing maximum velocity in older adults using inertial sensor system. PeerJ. 2020;8:e9329. 32704440 10.7717/peerj.9329 PMC7350916

[37] Sirhan B, Frid L, Kalron A. Is the dual-task cost of walking and texting unique in people with multiple sclerosis? J Neural Transm. 2018;125:1829–35. 30298276 10.1007/s00702-018-1939-4

[38] de Souza Fortaleza AC, Mancini M, Carlson-Kuhta P, King LA, Nutt JG, Chagas EF, et al. Dual task interference on postural sway, postural transitions and gait in people with Parkinson’s disease and freezing of gait. Gait Posture. 2017;56:76–81. 28521148 10.1016/j.gaitpost.2017.05.006 PMC5714292

[39] Forslund EB, Jorgensen V, Franzen E, Opheim A, Seiger A, Stahle A, et al. High incidence of falls and fall-related injuries in wheelchair users with spinal cord injury: A prospective study of risk indicators. J Rehabil Med. 2017;49:144–51. 28101557 10.2340/16501977-2177

